# Supplementary material for: Direct cost of inpatient wound dressing in South West Nigeria: A cross-sectional study
Source: PLoS One. 2025 Jun 25;20(6):e0326871. doi: 10.1371/journal.pone.0326871 (PMC12192046; doi:10.1371/journal.pone.0326871)
Supplement: Attachment — Submitted filename: Direct Cost of Wound Dressing.docx [file pone.0326871.s002.docx]

**Direct Cost of Wound Dressing: Hospitalized Patients Experience in South West Nigeria**

**Abstract**

**Line 42 – 43**

**…**the cost of dressing changes is not well studied…

What led you to look into this area of study now that you found it was not well studied? What was the problem? The problem statement is not clearly

**Line 46**

The title is “……hospitalised patient experiences……” but your method determines the costs using a cross-sectional design. Experiences- are qualitative, NOT quantity. Your design is different from your title. Please rephrase your title.

**Line 49**

Study period between May/July 2021

A stroke (/) means OR. Did you mean May to July (May – July)?

…One hundred ninety… Did you forget “and”

**Line 51**

You only mentioned ethical approval from hospitals. What about the University and patients? NB: Abstract is the face of your study!

**Line 47 and 50**

…(47) data were collected from hospitalised patients …

…(50) ….patients already discharged…

Do you hospitalise the patients discharged in Nigeria?

How did you include discharged patients in hospitalised patients?

**Lines 53 and 54**

These lines on your results are unclear as they have nothing to do with your study. Your aim is COSTS, not CAUSES OF WOUNDS. This abstract is misleading.

**Line 55**

Don’t start a sentence with a number

**Line 64**

**“…**hospitalised patients…” What about those discharged, as your inclusion criteria stated?

**Lines 81 -82**

Add reference

**Line 88**

You added a full stop before and after the reference- this is careless and irresponsible writing.

**Line 92**

“..6-folds..” please write the numbers below 10 in words

**Lines 94 and 95**

Harvard referencing was used incorrectly. Revise this sentence using the correct reference style.

**Line 104 - 107**

Long sentence; please rephrase

**Line 118**

A cross-sectional study is correct to investigate the costs. However, the title “hospitalised patient experiences” is not related to the study design utilised.

**Line 119**

The sampling method of the settings is given, but no sampling method of the participants

**Line 131 – 132**

Please specify your sampling method for these patients

**Line 150**

You have x2 “Sampling” in one sentence. This is grammatically incorrect. Please update

**Line 152 – 153**

Being discharged is not the same as being hospitalised; this is confusing

**Line 153**

“…not less than four weeks ago….”. That word “ago” doesn’t fit in the sentence - please revise and I suggest you download Grammarly to improve yours.

**Line 157**

Rephrase “Data collection instrument and procedure.”

A stroke (/) means OR

**Line 158**

What criteria were used to create the questionnaire?

**Line 161**

Remove “For the reliability test”

**Line 163**

Instead of writing “closer to one” write ……was 0.774

**Line 164**

What do you mean when you say it was for three months?

**Line 165**

“…for not less than four weeks…” This, again, is grammatically unclear. Please rephrase.

**Line 166**

“Written consent from each patient” Do you consider those discharged as patients? When will they be people again? How was the questionnaire administered to those at home?

**Line 168**

“…filled out…” – completed

**Line 168**

“..the research assistant filled out the questionnaire..”

How did you control bias if the researcher completed the questionnaire?

**Line 170 – 171**

How do these patients know the costs of the dressings, consumables, and materials?

**Line 172**

Data is plural therefore, data were NOT was

**Line 176 – 177**

This is a manuscript; data analysis must be clear as to what variables were categorised and which ones were presented as means and SD. You have written this as if you are writing a protocol.

**Line 181**

Remove “Also”

**Line 187 - 188**

Self-determination, confidentiality, and nonmaleficence must be explained as per the study manuscript, not as a protocol writing.

**Line 195 – 197**

Is a duplicate of Table 1

**Line 198 – 200, 204, 210**

Fig 1, 2, and 3 – “..the chart above..” Rewrite it considering that no chart is displayed above.

Figures are labelled on the bottom, NOT on top. Tables are labelled on top

**Page 8 Table 2**

Please add LINE numbers

**Table 2 Notes - Page 8**

Remove “also”

**Discussion**

“…Our findings…(line 3), please add reference.

**Discussion Line 6**

Study by (27)…….

Poor referencing and writing style

**Discussion Paragraph 2**

Line 3 “….a study by (30)….”

Fix your writing and reference

**Line 5 - same problem**

Paragraph 2, line 9

Harvard referencing used

**Line 10 –**

Who is 31 and 32? You have poor referencing and writing style.

Can you read those numbers in a sentence?

**Page 10, lines 2 - 4**

Harvard referencing

The same line is not related to your study. Why should you discuss the cause of the incidence of cancer while you are talking about wound costs?

**Line 24 – Paragraph 2 cause not related**

**Page 10 2^nd^ Paragraph**

Remove “Again” Rather use “In addition or Additionally”

NO REFERENCES ADDED IN PARAGRAPH 2

**Page 10 Last paragraph**

You said “several studies…” yet you only have one reference

**Page 10 - last paragraph Line 6**

You cannot say reported by (10) and (11)

Please use academic writing

**Page 10, lines 9-10**

“The costs will be higher when imported wound dressings are used…” Please justify the statement and give a reference

**Line 10 – 11**

“..Some scholars argue…” Please give reference of those scholars

**Page 11, Line 1**

Please add reference

**Page 11, Line 4**

“…ALSO…” Change to academic writing

**Line 4**

“….34 conducted…?”

Who is 34? Please use academic writing and the correct referencing style

**Page 11, line 6**

“…34 study….” Please correct

**Line 7**

“ ..Again..” Please use academic writing

**Line 8**

Add reference

**Line 9**

“…researchers….”

Add reference

**Page 11, paragraph 2, line 1**

“…A scoping review by 7…”?????????

In the same paragraph, you used “Fig then Figure”

Do you want to abbreviate it or write it in full? Be consistent

**Page 11, last line**

Started a sentence with (41)????

**Page 12, line 1**

“In (40)…” ?????

**Page 12, paragraph 1**

Please add references

**Limitations**

- “Recall bias”

Is this what you call patient experiences?

This was more a record review than experience not even a cross-sectional

**Paragraph 2**

Why do you need “proper documentation” if you are doing a cross-sectional study of the patients?

Again, you mentioned that you utilised the Nurse to get information regarding wound dressings. Was this study not for patients? why nurses were needed

**Acknowledgement**

- What were the nurses' duties? I didn’t hear of their involvement in the methodology section.
