# Supplementary material for: Direct cost of inpatient wound dressing in South West Nigeria: A cross-sectional study
Source: PLoS One. 2025 Jun 25;20(6):e0326871. doi: 10.1371/journal.pone.0326871 (PMC12192046; doi:10.1371/journal.pone.0326871)
Supplement: Attachment — Submitted filename: Plos one_review outcome.docx [file pone.0326871.s003.docx]

**Overview**

First, I commend the authors for the efforts and rigour invested into this study. This is a cross-sectional study entitled “Direct Cost of Wound Dressing: Hospitalized Patients Experience in South West Nigeria” sought to determine the cost implications of wound dressing across wound aetiology (sic) among hospitalized patients. According to the authors,” 34.2% of the respondents had road traffic accidents, followed by cancers, 22.6%, and surgical wound infections, 16.8%. Most patients were involved in daily (41.6%) or alternate-day (38.4%) wound dressing. 53.2% of the respondents earn less than US$30 per month, 34.7% earn between US$30 - US$60, while only 3.2% earn more than US$ 120 per month. Also, 55.7% require 1-5 moderate or significant dressing packs per week. 75% had wound care paid for by relatives. The average burn injuries cost of wound dressing per week is estimated to be $8.42, while falls ($5.31), occupational injuries ($3.77), gunshot injuries ($3.74), and road traffic accidents ($3.49). The average cost of hospitalization for burn injuries per week was estimated to be $22.35, while for falls, road traffic accidents, and surgical wound infections, was $19.58, $19.32, and $18.37, respectively.” Authors concluded thus “The cost requirements for prosperous wound dressing place a high financial burden on hospitalized patients in Nigeria. There is a need to scale Nigeria's health insurance database to include the low socioeconomic class”

**Areas of improvements**

The study possesses important data for the South Africa populace. In addition, the study was rigorously conducted and manuscript well-written. However, a few minor revisions are required to improve coherence and readability.

*Title*

The study title needs to be rephrased. The use of “experience” suggests that the study employed interpretivist paradigm, whereas in practice, the authors adopt positivism. Therefore, , based on first impression, title is misleading.

*Introduction*

I would acknowledge similar previous studies in Nigeria and highlight a few of their weakness/gaps and how the present study would fill the gap(s).

*Misuse of aetiology*

Many times, the authors misused the term “aetiology”. I would prefer the use of causes or mechanism of injuries in many instances where the authors used ‘aetiology” which is an umbrella term referring to the cause (i.e. all the causes) of a disease/injury including the mechanism of injury.

*Method*

Design: The checklist used to guide the study was not specified.

*Discussion*

Whereas authors compared the cost data with other similar data in other climes, I would first focus on comparing the data from the present study with similar Nigerian studies. This will allow the readers to decide if the data presented in this current study is an outlier from other similar Nigeria study and if so, what could the reason (changing times?). Second, I would then compare the data with similar data in other climes, bearing in mind the Nigerian average. Kindly revise as appropriate.

**Decision**

I recommend the publication of the manuscript following minor revision.
