# Supplementary material for: Direct cost of inpatient wound dressing in South West Nigeria: A cross-sectional study
Source: PLoS One. 2025 Jun 25;20(6):e0326871. doi: 10.1371/journal.pone.0326871 (PMC12192046; doi:10.1371/journal.pone.0326871)
Supplement: Attachment — Submitted filename: Response to Reviewers Comments.docx [file pone.0326871.s005.docx]

**Response to Reviewers' Comments**

| **Section** | **Comments** | **Response** |
| --- | --- | --- |
| Title | The study title needs to be rephrased. The use of “experience” suggests that the study employed an interpretivist paradigm, whereas in practice, the authors adopt positivism. Therefore, based on the first impression, the title is misleading. | The word experience was removed. Title rephrased:  Direct cost of inpatient wound dressing in South West Nigeria: A cross-sectional study |
| Introduction | I would acknowledge similar previous studies in Nigeria and highlight a few of their weakness/gaps and how the present study would fill the gap(s). | There is a paucity of studies that categorically investigate the cost of inpatient wound dressing in Nigeria and across Sub-Saharan Africa.  The gap in most studies is that the cost of wound dressing is seen as a room and board rate. The cost is mostly part of the medical or surgical procedural cost. This is the gap that this current study has filled  However, few previous studies were reviewed, and their contribution, strengths, and weaknesses were already discussed in the introduction and the discussion section |
| Misuse of Aetiology | Many times, the authors misused the term “aetiology”. I would prefer the use of causes or mechanism of injuries in many instances where the authors used ‘aetiology” which is an umbrella term referring to the cause (i.e. all the causes) of a disease/injury including the mechanism of injury. | The authors considered the word aetiology appropriate because the wound in this context has many causes, i.e., road traffic accident, fall, cancer, surgical site infection, etc. |
| Method | Design: The checklist used to guide the study was not specified. | A self-designed questionnaire was used to collect data via a researcher-administered questionnaire. The questionnaire design was based on a literature review on the subject and the researcher's previous wound care study experience  Variables in the questionnaire were mentioned in the instrument for the data collection section of the method:  Variables of interest included wound aetiology, frequency of wound dressing per week, type and number of dressing consumables used per week, monthly income, cost of wound dressing materials per week, and payment for wound care consumables |
| Discussion | Whereas authors compared the cost data with other similar data in other climes, I would first focus on comparing the data from the present study with similar Nigerian studies. This will allow the readers to decide if the data presented in this current study is an outlier from other similar Nigeria study and if so, what could the reason (changing times?). Second, I would then compare the data with similar data in other climes, bearing in mind the Nigerian average. Kindly revise as appropriate. | In the second segment of the discussion, the authors compared the cost of wound dressing to findings elsewhere chronologically  The first was to compare the cost with the cost of wound dressing in previous studies within Sub-Saharan Africa, with Nigeria inclusive. Few studies conducted in a similar setting- southwest Nigeria were reviewed. The gap remains that most studies did not specifically track the cost of dressing, and therefore were not included in the literature review  Followed by a comparison with the cost of care in another region with a similar Human Development Index. Costs were compared with the cost of dressing in previous studies in Asia, particularly in India, Iran  Costs were also compared with costs reported in systematic reviews across the world  Generally, studies tracking the cost of wound dressing are sparse worldwide, and this is the current focus of the researchers |
| **Second Reviewer** |  |  |
| Abstract | **Line 42 – 43**  **…**the cost of dressing changes is not well studied…  What led you to look into this area of study now that you found it was not well studied? What was the problem? The problem statement is not clearly | It has not been well studied because few studies track the cost of wound dressing. Wound dressing is an important part of wound care protocol. Most of the cost of wound dressing is summed into the surgical procedural cost. The thrust of this study was to fill this gap |
| Title | The title is “……hospitalised patient experiences……” but your method determines the costs using a cross-sectional design. Experiences- are qualitative, NOT quantity. Your design is different from your title. Please rephrase your title. | Thank you  The title has been rephrased as:  Direct cost of inpatient wound dressing in South West Nigeria: A cross-sectional study |
|  | **Line 49**  Study period between May/July 2021  A stroke (/) means OR. Did you mean May to July (May – July)?  …One hundred ninety… Did you forget “and” | Correction made  It is May to July |
|  | **Line 51**  You only mentioned ethical approval from hospitals. What about the University and patients? NB: Abstract is the face of your study!  **Line 47 and 50**  …(47) data were collected from hospitalised patients …  …(50) ….patients already discharged…  Do you hospitalise the patients discharged in Nigeria?  How did you include discharged patients in hospitalised patients? | University added  It was actually because of the limited word count for the abstract  O.k.  We meant patients about to be discharged. In Nigeria, patients do not leave immediately after they are discharged. There is discharge teaching  Best put as “Patient about to be discharged”. Thank you |
|  | **Lines 53 and 54**  These lines on your results are unclear as they have nothing to do with your study. Your aim is COSTS, not CAUSES OF WOUNDS. This abstract is misleading. | We considered it important to briefly mention the causes before reporting the cost, which is the main result  If you read further, the result section mostly reported the cost of wound dressing |
|  | **Line 55**  Don’t start a sentence with a number | Agreed and corrected- Over 50% |
|  | **Line 64**  **“…**hospitalised patients…” What about those discharged, as your inclusion criteria stated? | As stated above, they are still in the ward. They were about to be discharged |
|  | **Lines 81 -82**  Add reference | References added |
|  | You added a full stop before and after the reference- this is careless and irresponsible writing. | It is the EndNote software. Correction made |
|  | **Line 92**  “..6-folds..” please write the numbers below 10 in words | Corrected. “Six-folds” |
|  | **Lines 94 and 95**  Harvard referencing was used incorrectly. Revise this sentence using the correct reference style. | Vancouver  Correction done |
|  | **Line 104 - 107**  Long sentence; please rephrase | Short sentences separated by a full stop. Kindly recheck |
|  | **Line 118**  A cross-sectional study is correct to investigate the costs. However, the title “hospitalised patient experiences” is not related to the study design utilised. | Title already rephrased above |
|  | **Line 119**  The sampling method of the settings is given, but no sampling method of the participants | The respondents were recruited via convenience sampling  This was discussed under “sample and sampling strategy.” |
|  | **Line 131 – 132**  Please specify your sampling method for these patients | The respondents were recruited via convenience sampling  This was discussed under “sample and sampling strategy.” |
|  | **Line 150**  You have x2 “Sampling” in one sentence. This is grammatically incorrect. Please update | The word sampling was not written twice in a sentence. Kindly recheck, please. |
|  | **Line 152 – 153**  Being discharged is not the same as being hospitalised; this is confusing | Had discharged change to “about to be discharged” |
|  | **Line 153**  “…not less than four weeks ago….”. That word “ago” doesn’t fit in the sentence - please revise and I suggest you download Grammarly to improve yours. | The word “ago” was deleted  Our university subscribes to Grammarly  The English editing will be done before resubmission |
|  | **Line 157**  Rephrase “Data collection instrument and procedure.”  A stroke (/) means OR | O.k. Correction done |
|  | **Line 158**  What criteria were used to create the questionnaire? | The questionnaire was designed based on a literature review on the cost of wound dressing and the researcher's previous experience in wound care and cost |
|  | **Line 161**  Remove “For the reliability test”  **Line 163**  Instead of writing “closer to one” write ……was 0.774 | Yes. Thank you.  Removed  O.K. Correction done |
|  | **Line 164**  What do you mean when you say it was for three months? | Data collected between May to July  One month per centre |
|  | **Line 165**  “…for not less than four weeks…” This, again, is grammatically unclear. Please rephrase. | **Corrected as**  “” had been admitted for four weeks |
|  | **Line 166**  “Written consent from each patient” Do you consider those discharged as patients? When will they be people again? How was the questionnaire administered to those at home? | As stated above  They were inpatients about to be discharged |
|  | **Line 168**  “…filled out…” – completed | Done |
|  | **Line 168**  “..the research assistant filled out the questionnaire..”  How did you control bias if the researcher completed the questionnaire? | The research assistants were clinical nurses. They were also trained on the purpose of the study and how to complete the questionnaire accurately without bias  The completed questionnaires were cross-checked by the principal investigator |
|  | **Line 170 – 171**  How do these patients know the costs of the dressings, consumables, and materials? | Firstly, they were adult and oriented. They gave the cost of materials, validated by the attached receipt in the file  If bought in the hospital, we also confirm the price in the hospital Central Sterile Storage Department (CSSD) and pharmacy |
|  | **Line 172**  Data is plural therefore, data were NOT was | Corrected |
|  | **Line 176 – 177**  This is a manuscript; data analysis must be clear as to what variables were categorised and which ones were presented as means and SD. You have written this as if you are writing a protocol. | Monthly income and wound characteristics (aetiologies, frequency of wound dressing, number and type of dressing pack used) were presented in figures, while the costs of wound dressing were presented in mean and standard deviation |
|  | **Line 181**  Remove “Also” | O.K. Thank you |
|  | **Line 187 - 188**  Self-determination, confidentiality, and nonmaleficence must be explained as per the study manuscript, not as a protocol writing. | They were first listed, and then the next statement explained what was done to ensure the ethical principles  **“**The patient’s identifying information was not included. Patients were told of their right to participate or withdraw from the study at any time without being victimized” |
|  | **Line 195 – 197**  Is a duplicate of Table 1 | Table 1. No duplicate |
|  | **Line 198 – 200, 204, 210**  Fig 1, 2, and 3 – “..the chart above..” Rewrite it considering that no chart is displayed above.  Figures are labelled on the bottom, NOT on top. Tables are labelled on top | O.K. |
|  | **Page 8 Table 2**  Please add LINE numbers  **Table 2 Notes - Page 8**  Remove “also” |  |
| **Discussion** | “…Our findings…(line 3), please add reference. | From other parts of Nigeria: |
|  | **Discussion Line 6**  Study by (27)…….  Poor referencing and writing style | Onyemaechi et al  Added |
|  | **Discussion Paragraph 2**  Line 3 “….a study by (30)….”  Fix your writing and reference | Lawal et al  Added |
|  | **Line 5 - same problem**  Paragraph 2, line 9  Harvard referencing used  **Line 10 –**  Who is 31 and 32? You have poor referencing and writing style.  Can you read those numbers in a sentence? | Singh (32)  Mohammed et al and Shaaban et al  Added |
|  | **Page 10, lines 2 - 4**  Harvard referencing  The same line is not related to your study. Why should you discuss the cause of the incidence of cancer while you are talking about wound costs? | Extended discussion on cancer removed. Page 10 |
|  | **Page 10 2^nd^ Paragraph**  Remove “Again” Rather use “In addition or Additionally”  NO REFERENCES ADDED IN PARAGRAPH 2 | “Again” deleted  In addition  Most discussion on cancer were removed |
|  | **Page 10 Last paragraph**  You said “several studies…” yet you only have one reference | References added |
|  | **Page 10 - last paragraph Line 6**  You cannot say reported by (10) and (11)  Please use academic writing | Reported removed |
|  | **Page 10, lines 9-10**  “The costs will be higher when imported wound dressings are used…” Please justify the statement and give a reference  **Line 10 – 11**  “..Some scholars argue…” Please give reference of those scholars | References added  References added |
|  | **Page 11, Line 1**  Please add reference | References added |
|  | **Page 11, Line 4**  “…ALSO…” Change to academic writing | Done |
|  | **Line 4**  “….34 conducted…?”  Who is 34? Please use academic writing and the correct referencing style | Done |
|  | **Page 11, line 6**  “…34 study….” Please correct | Done |
|  | **Line 7**  “ ..Again..” Please use academic writing | Done |
|  | **Line 8**  Add reference | Done |
|  | **Line 9**  “…researchers….”  Add reference | Done |
|  | **Page 11, paragraph 2, line 1**  “…A scoping review by 7…”?????????  In the same paragraph, you used “Fig then Figure”  Do you want to abbreviate it or write it in full? Be consistent | Handayani et al  Added  Correction done |
|  | **Page 11, last line**  Started a sentence with (41)???? | Correction done |
|  | **Page 12, line 1**  “In (40)…” ????? | Correction done |
|  | **Page 12, paragraph 1**  Please add references | References added |
| **Limitation** | - “Recall bias”   Is this what you call patient experiences?  This was more a record review than experience not even a cross-sectional | It is cross-sectional: Researcher-administered questionnaire was used to collect data  The patient's case file was only checked to identify receipt of procurements if needed  Paragraph indicative information related to the chart review in the limitation has been deleted |
| **Acknowledgement** | **Paragraph 2**  Why do you need “proper documentation” if you are doing a cross-sectional study of the patients?  Again, you mentioned that you utilised the Nurse to get information regarding wound dressings. Was this study not for patients? why nurses were needed   - What were the nurses' duties? I didn’t hear of their involvement in the methodology section. | Information on proper documentation deleted  No information was asked from the nurses regarding wound dressing  Nurses in the ward mainly assisted in identifying patients about to be discharged  Nurses in the ward mainly assisted in identifying patients about to be discharged  However, the research assistants were nurses  These are mentioned under the procedure for data collection |
| **Editor-3** | Please include a copy of Tables number 5 and 3 which you refer to in your text on page 10 and 12. | Please, it is Table 2 not 5  Not Table 3, the information is deleted |
| **Editor-4** | 4. Please review your reference list to ensure that it is complete and correct. If you have cited papers that have been retracted, please include the rationale for doing so in the manuscript text, or remove these references and replace them with relevant current references. Any changes to the reference list should be mentioned in the rebuttal letter that accompanies your revised manuscript. If you need to cite a retracted article, indicate the article’s retracted status in the References list and also include a citation and full reference for the retraction notice. | To the best of my knowledge, no retracted articles are cited in the manuscript  Additional references were made. All journal articles cited within the manuscript are listed in Vancouver style in the reference list ( at the end of the manuscript) |
|  | Files uploaded to PACE and downloaded  Kolawole.ogundeji@gmail.com |  |
